# Coupled high-throughput functional screening and next generation sequencing for identification of plant polymer decomposing enzymes in metagenomic libraries

**DOI:** 10.3389/fmicb.2013.00282

**Published:** 2013-09-23

**Authors:** Mari Nyyssönen, Huu M. Tran, Ulas Karaoz, Claudia Weihe, Masood Z. Hadi, Jennifer B. H. Martiny, Adam C. Martiny, Eoin L. Brodie

**Affiliations:** ^1^Ecology Department, Earth Sciences Division, Lawrence Berkeley National LaboratoryBerkeley, CA, USA; ^2^The Joint BioEnergy InstituteEmeryville, CA, USA; ^3^Sandia National LaboratoriesLivermore, CA, USA; ^4^Department of Ecology and Evolutionary Biology, University of California IrvineIrvine, CA, USA; ^5^Department of Earth System Science, University of California IrvineIrvine, CA, USA; ^6^Department of Environmental Science, Policy and Management, University of CaliforniaBerkeley, CA, USA

**Keywords:** functional metagenomics, carbon cycling, trait-based modeling, gene annotation, microbial communities, decomposers, metagenomics, enzyme activity

## Abstract

Recent advances in sequencing technologies generate new predictions and hypotheses about the functional roles of environmental microorganisms. Yet, until we can test these predictions at a scale that matches our ability to generate them, most of them will remain as hypotheses. Function-based mining of metagenomic libraries can provide direct linkages between genes, metabolic traits and microbial taxa and thus bridge this gap between sequence data generation and functional predictions. Here we developed high-throughput screening assays for function-based characterization of activities involved in plant polymer decomposition from environmental metagenomic libraries. The multiplexed assays use fluorogenic and chromogenic substrates, combine automated liquid handling and use a genetically modified expression host to enable simultaneous screening of 12,160 clones for 14 activities in a total of 170,240 reactions. Using this platform we identified 374 (0.26%) cellulose, hemicellulose, chitin, starch, phosphate and protein hydrolyzing clones from fosmid libraries prepared from decomposing leaf litter. Sequencing on the Illumina MiSeq platform, followed by assembly and gene prediction of a subset of 95 fosmid clones, identified a broad range of bacterial phyla, including Actinobacteria, Bacteroidetes, multiple Proteobacteria sub-phyla in addition to some Fungi. Carbohydrate-active enzyme genes from 20 different glycoside hydrolase (GH) families were detected. Using tetranucleotide frequency (TNF) binning of fosmid sequences, multiple enzyme activities from distinct fosmids were linked, demonstrating how biochemically-confirmed functional traits in environmental metagenomes may be attributed to groups of specific organisms. Overall, our results demonstrate how functional screening of metagenomic libraries can be used to connect microbial functionality to community composition and, as a result, complement large-scale metagenomic sequencing efforts.

## Introduction

The structure and functionality of microbial communities plays a key role in the resilience and functioning of ecosystems. Understanding the relationship between microbial contributions to ecosystem processes may be essential to predicting how ecosystems respond to environmental change (Loreau et al., 2001; Allison and Martiny, 2008; Allison, 2012). However, relating the composition of mostly uncultured microbial communities to functional traits, and further to ecosystem functioning, is challenging.

Shotgun sequencing of metagenomic DNA isolated from environmental microbial communities has rapidly advanced our understanding of their functional diversity, evolution and role in ecosystem functioning (Venter et al., 2004; Tringe et al., 2005; Denef and Banfield, 2012; Fierer et al., 2012; Wrighton et al., 2012). Reconstructing genomes from environmental sequence data has been particularly valuable in studying the metabolic potential housed in the genomes of uncultured microbes (Tyson et al., 2004; Mackelprang et al., 2011; Iverson et al., 2012). In the meantime, large scale sequencing efforts geared toward the prediction of microbial processes and their response to environmental change are currently under way (e.g., Earth Microbiome Project; Great Prairie Grand Challenge; MetaSoil Project).

Despite the increased generation of sequence data, a challenge remains in providing accurate functional predictions for the increasing number of novel genes and gene families and in confirming functions for environmental homologs of previously characterized genes. For this reason, assays that provide biochemical confirmation of activity are necessary to improve *in silico* predictions, many of which are purely hypothetical or potentially inaccurate and misleading (Schnoes et al., 2009; Prakash and Taylor, 2012). Furthermore, in addition to improving genome annotation, biochemical activity assays of novel genes improve our general understanding of microbial functional traits. Such traits are often phylogenetically conserved among microbial taxa and/or are correlated with one another (e.g., Martiny et al., 2012; Berlemont and Martiny, 2013; Zimmerman et al., 2013). For this reason, biochemically-confirmed gene functions can provide more accurate insights into the metabolic potential of a microbial community, its response to environmental change and the potential impacts on ecosystem processes.

Direct cloning and activity-based screening of metabolic potential of uncultured microbes in a heterologous expression host provides direct linkages between genes, ecologically important metabolic traits and microbial phylogeny. Because no *a priori* sequence information is required, this phenotypic characterization approach provides direct confirmation of activity and/or substrate specificity for either previously characterized or novel genes and gene families (e.g., Mirete et al., 2007; Nacke et al., 2011; Chow et al., 2012). As a result, it has great potential to overcome some of the current *de novo* annotation pitfalls that limit interpretation of environmental sequence data.

To date, the function-driven characterization of metagenomic libraries has mainly focused on traits of economical or biomedical value such as bioconversion of cellulosic materials (Hu et al., 2008; Pang et al., 2009; Jiang et al., 2011; Nacke et al., 2011), contaminant biodegradation (Ono et al., 2007; Brennerova et al., 2009) and antibiotic synthesis and resistance (Chung et al., 2008; Allen et al., 2009; McGarvey et al., 2012). However, this approach can also provide insights into the biogeochemistry and ecology of natural environments, such as pathogen-suppression in soils (van Elsas et al., 2008), evolution of contaminant degradation pathways (Slámová et al., 2009), communication within microbial populations (Chung et al., 2008; Riaz et al., 2008) and microbial colonization of surfaces (Yoon et al., 2013). These results complement environmental sequencing efforts and can ultimately lead to better understanding of ecosystem functioning.

However, the success of functional mining of metagenomic libraries is dependent on the stochastic nature of DNA isolation, cloning and expression. Although this is important in linking novel enzyme genes to biochemically confirmed function, it limits our ability to fully exploit the potential of activity-based screening in functional annotation. The frequency of target genes in microbial genomes is low, e.g., less than two bacterial cellulolytic glycoside hydrolases per microbial genome (Berlemont and Martiny, 2013). Consequently, invariably large numbers of clones must be screened in order to adequately sample a community for functional annotation at a rate that is consistent with that of metagenomic sequence data acquisition. Thus, a key to the success is increased analytical throughput as well as sensitive and specific screening assays.

Phenotype detection using solid media based screening assays has the capacity to screen large numbers of clones and thus increase the analytical throughput. It has been extensively used to mine novel enzymes and bioactive compounds from metagenomic libraries (e.g., Pang et al., 2009; Craig et al., 2010). For instance, solid media assays enabled Tasse et al. (2010) to screen 200,000 clones against 7 primary and 16 secondary substrates per week to identify activities involved in dietary fiber breakdown in the human gut microbiome. Though cost-effective and highly scalable in throughput, such solid media based assays exhibit low signal-to-noise ratios in part due to the diffusion of reaction products (Sharrock, 1988). This results in decreased detection sensitivity and data with limited quantitative value. As an alternative, solution-based assays are amenable to automated liquid-handling systems, and coupled with the direct detection of chromogenic or fluorescent substrate transformations, can be used to increase screening throughput, assay reproducibility, and sensitivity. Yet, few reports on automated solution-based screening assays have been published to date. Mewis et al. (2011) recently described a high-throughput screening assay for detecting cellulase activity in metagenomic libraries. Although the authors achieved a daily throughput of 38,400 clones by using one labeled substrate, 384 well microplate format, and automated liquid handling, further improvements in analysis throughput, sensitivity and specificity are needed for functional metagenomics to keep pace with sequence data generation.

In this study, our aim was to further increase throughput, sensitivity and specificity of functional screening assays for characterization of genes and activities contained in environmental metagenomes. In order to achieve this we combined (1) the concurrent use of multiple substrates with different detection modalities (e.g., colorimetric or fluorescence), (2) pooling and expansion of clones in solution-based assays using automated liquid handling, and (3) genetic modification of the expression host and induction of vector copy number. The throughput of the developed solution and agar-based assays is 12,160 clones per day with simultaneous screening for 14 different activities involved in cellulose, hemicellulose, chitin, starch and lignin decomposition, organic phosphate mineralization and protein hydrolysis in a total of 170,240 assays. To demonstrate the utility of these assays in characterizing the targeted activities from uncultured environmental microbes, we screened seven fosmid libraries constructed from decomposing leaf litter. We then identified the genetic basis for the observed enzymatic activities in a selected subset of clones using next generation sequencing on the Illumina MiSeq platform. Finally, using tetranucleotide frequency (TNF) binning of assembled sequences, we propose a means to connect functional traits detected on distinct fosmid inserts to their likely genomic origin. Overall, our results demonstrate how functional screening of metagenomic libraries can be used to connect microbial functionality to community composition and, as a result, complement large-scale metagenomic sequencing efforts.

## Materials and methods

### Bacterial strains, plasmids, enzymes, and environmental samples

*Escherichia coli* strains BW25141/pKD13, BW25113/pKD46, and BT340 were obtained from the *E. coli* Genetic Stock Center (Yale University, New Haven, CT, USA). Phage resistant *E. coli* EPI300-T1^R^ used as a parental strain for knockouts was obtained from EpiCenter Biotechnologies (Madison, WI, USA). The strains were grown on LB with 100 μg ml^−1^ ampicillin, 50 μg ml^−1^ kanamycin or without antibiotic selection. Plasmid DNA was isolated from the BW25141, BW25113, and BT340 strains with the Qiaprep Spin Miniprep Kit (Qiagen, Germantown, MD, USA). Purified enzymes were obtained from commercial suppliers (Table S2). Samples for fosmid library construction were collected from decomposing leaf litter at a whole ecosystem manipulation site in annual grassland at Loma Ridge, CA, USA (33° 44′ N, 117° 42′ W) (Potts et al., 2012; Allison et al., 2013). The samples were collected from control plots with ambient precipitation and ambient nitrogen deposition (denoted XX) in December 2010, February 2011, and March 2012 and from ambient precipitation and nitrogen enrichment plots (denoted XN) in February 2011. After sampling, litter was ground in a coffee grinder and immediately frozen at −80°C.

### Gene disruption

**Figure 1:**
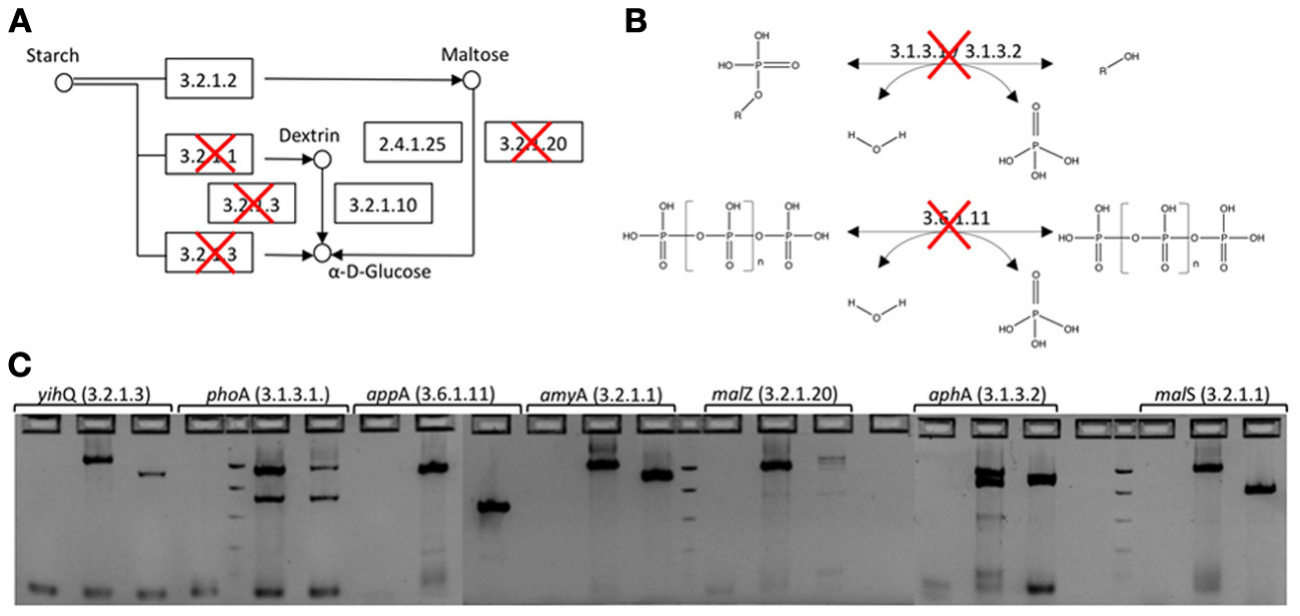
**In-frame deletion of genes involved in starch degradation and phosphate hydrolysis from the *E. coli* EPI300-T1^R^ expression host. (A)** Starch degradation pathway. Deleted genes have been crossed out. **(B)** Phosphatase catalyzed reactions that were knocked out. **(C)** Agarose gel electorophoresis of PCR amplicons before and after gene deletion. For each gene, the first well is PCR negative control, second is wild type *E. coli* EPI300-T1^R^ and third is *E. coli* EPI300-T1^R^ after gene deletion (EC 3.2.1.3 α-glucosidase; EC 3.1.3.1 alkaline phosphatase; EC 3.6.1.11 phosphoanhydride phosphohydrolase; EC 3.2.1.1 cytoplasmic or periplasmic α-amylase; EC EC 3.2.1.20 maltodextrin glucosidase; EC 3.1.3.2 acid phosphatase).

*E. coli* EPI300-T1r, used as the host strain in the functional screening, carries genes involved in both amylose and phosphate metabolism, i.e., activities that could increase background signal in screening assays for starch-hydrolyzing enzymes or phosphatases. In order to reduce these wild type enzymatic activities and to increase signal-to-noise ratio and sensitivity of the starch and phosphatase assays, seven genes involved in starch degradation or phosphate mineralization were deleted from *E. coli* EPI300-T1^R^ genome using in-frame gene disruptions (Figures 1 and S1). The seven gene specific knockouts were created with the one-step gene inactivation method developed by Datsenko and Wanner (2000) (Figure S1, Table S1). Gene disruption cassettes were generated by PCR using the N-terminal and C-terminal deletion primers described in Baba et al. (2006) (Table S1). PCR reaction mixes (50 μl) contained 1 × Ex Taq Buffer, 0.2 mM of each deoxynucleoside triphosphate, 50 pmol of each primer, 1.25 U TaKaRa Ex Taq DNA polymerase (TaKaRa Bio Inc., Otsu, Shiga, Japan) and 12.7 ng of pKD13 DNA as a template. Thermocycling parameters were 5 min at 95°C, followed by 30 cycles of 30 s at 95°C, 30 s at 59°C and 2 min at 72°C and a final extension of 2 min at 72°C. Amplification products were DpnI treated for 2 h at 37°C to digest plasmid DNA used as a template for PCR, electrophoresed on 2% E-Gels (Invitrogen, Carlsbad, CA, USA) and gel purified using QIAquick Gel Extraction Kits (Qiagen).

The *E. coli* EPI300-T1^R^ parental strain and subsequent gene deletion strains were grown on super optimal broth (SOB, 2% peptone, 0.5% yeast extract, 10 mM NaCl, 2.5 mM MgCl_2_, 10 mM MgSO_4_) to OD_600_ of 0.6–0.8 and made electrocompetent by rinsing three times with ice-cold 10% glycerol. The cells were then transformed with the pKD46 Red expression plasmid by electroporation using a Gene Pulser™ (Bio-Rad Laboratories, Hercules, CA, USA) at 1.8 kV, 25 μF, and 200 Ω. Transformants were selected on LB agar containing 100 μg ml^−1^ ampicillin at 30°C overnight.

In order to induce the Red system and prepare cells for transformation, the pKD46 transformants were grown at 30°C on SOB containing 100 μg ml^−1^ ampicillin and 0.3% L-arabinose to OD_600_ of 0.3–0.4. The temperature was increased to 37°C for 45 min and electrocompetent cells were prepared as described above. The cells were then transformed by electroporation with 1–2 μg of gel purified gene disruption cassette, suspended in super optimal broth with catabolic repressor (SOC) containing 0.3% L-arabinose and incubated at 37°C for 2 h. Gene deletion mutants were selected on Luria-Bertani (LB) agar containing 50 μg ml^−1^ kanamycin at 37°C overnight.

The correct insertion of the gene disruption cassette was verified by PCR amplification using test primers described in Datsenko and Wanner (2000) and locus-specific primers designed in this study (Table S1). The PCR components were 1 × Ex Taq Buffer, 0.2 mM of each deoxynucleoside triphosphate, 50 pmol of each primer, 1.25 U TaKaRa Ex Taq DNA polymerase and 1 μl of cell suspension. Thermocycling consisted of 5 min initial denaturation at 95°C followed by 35 cycles of 30 s at 95°C, 30 s at primer specific annealing temperature and 2 min at 72°C.

In order to eliminate the antibiotic resistance gene from the gene deletion mutants, the transformants were grown on SOB at 37°C and transformed by electroporation with the pCP20 FPL recombinase helper plasmid. Transformants were selected on LB agar containing 100 μg ml^−1^ ampicillin at 30°C overnight after which they were streaked on LB agar without antibiotics and incubated overnight at 43°C to cure from pCP20.

Deletion of the knockout cassette was verified with test primers and locus-specific primers as described above. The gene deletion mutants were also grown on LB with 100 μg ml^−1^ ampicillin or 50 μg ml^−1^ kanamycin to test for the loss of antibiotic resistance cassette. Wild type parental strains were used as controls in both PCR and growth tests.

### Fosmid library construction

DNA from litter samples collected in December 2010 and February 2011 was extracted as follows: 0.5 grams of litter was mixed with 7–10 ml of denaturing buffer [4 M guanidine thiocyanate, 10 mM TrisHCl (pH 7.6), 1 mM EDTA and 0.5% β-mercaptoethanol]. Samples were homogenized twice for 30 s at 25 mHz using a Tissue-Tearor (BioSpec Products, Bartlesville, OK, USA) with a 12 mm tip. Subsequently the mixture was flash frozen in liquid nitrogen and thawed for 5 min at 60°C. The freeze-thaw process was repeated three times. The homogenate was then mixed with 20 ml of 60°C extraction buffer [5% hexadecyltrimethylammonium bromide (CTAB), 1 M NaCl, 0.25 M phosphate buffer pH 8] and the mixture was incubated in a hybridization oven for 40 min at 60°C with rotation at 15 rpm. The extraction was repeated again with 10 ml of extraction buffer for 10 min at 60°C. The sample was then extracted with phenol:chloroform:isoamyl alcohol (25:24:1) and DNA was precipitated with 0.6 volumes of isopropanol, washed once with 70% ethanol and then re-suspended in TE buffer (10 mM Tris, 1 mM EDTA pH 8.0).

Litter samples collected in March 2012 did not yield sufficient amount of DNA using the extraction method described above. Therefore the samples were extracted using a combination of enzymatic and chemical cell lysis followed by hot phenol treatment and physical shearing. 0.4 g of litter was frozen in liquid nitrogen, homogenized using a Tissuelyzer (Qiagen) at 25 Hz for 30 s, mixed with 3 ml of extraction buffer [100 mM Tris-HCl (pH 8.0), 100 mM EDTA (pH 8.0), 100 mM NaPO_4_ (pH 8.0), 1.5 M NaCl, 1% CTAB, and lysozyme (10 mg ml^−1^)] and incubated at 37°C for 30 min. Proteinase K (20 mg ml^−1^) and SDS (10%) were added to the homogenate and incubation was continued at 55°C for 45 min followed by addition of 3 ml of phenol:chloroform:isoamylalcohol (25:24:1) and additional incubation at 60°C for 30 min. During all incubations, the samples were inverted manually every 15 min. Next, aqueous supernatant was collected, the remaining pellet was mixed with Lysing matrix E (MP Biomedicals Inc., Solon, OH, USA) and 3 ml of extraction buffer, and the sample was mixed by vortexing for 4 min at room temperature. Aqueous supernatant was removed, 3 ml of extraction buffer was added and the samples were treated for 15 s at 4 m s^−1^ in FastPrep-24 (MP Biomedicals Inc.). After cell lysis, all supernatants were extracted with chloroform:isoamyl alcohol (24:1). DNA was precipitated overnight with 2 volumes of 30 % polyethylene glycol-salt solution (1.6 M NaCl, 30% PEG 6000), washed with 70% ethanol twice and re-suspended in TE.

Four replicate crude extracts were pooled for each fosmid library and concentrated using Amicon Ultra 100 K filters (Millipore, Billerica, MA, USA). DNA fragments between 36 and 48 kb were then size-selected with a 0.8% agarose gel run at 35 V and 4°C for 18 h followed by excision of DNA fragments of the appropriate size. The size-selected DNA was recovered from agarose plugs by electroelution (GeneCAPSULE, G Biosciences, St. Louis, MO, USA) and concentrated on Amicon Ultra 100 K filters.

Fosmid libraries were constructed from the size-selected high molecular weight DNA with the CopyControl Fosmid Library Production Kit (Epicentre Biotechnologies, Madison, WI, USA) according to manufacturer's protocol with the following exceptions. End-repaired DNA was concentrated on Amicon Ultra 100 K filters (Millipore) before proceeding to ligation, and the *E. coli* host strain used was the gene disrupted mutant described above (*E. coli* EPI300-T1^R^ Δ*yih*Q Δ *pho*A Δ *app*A Δ *amy*A Δ *mal*Z Δ *aph*A Δ *mal*S). Duplicate libraries were prepared from each sampling time point with the exception of the nitrogen enrichment samples (XN) that were collected in February 2011 (Table 1). A control library for quantifying non-specific background activity was constructed from kit supplied control DNA.

**Table 1:** **Fosmid libraries constructed and screened in this study**.

**Library**	**Dec10_01XX**	**Dec10_08XX**	**Feb11_01XX**	**Feb11_08XX**	**Feb11_16XN**	**Mar12_01XX**	**Mar12_08XX**
Sampling time	December 2010	December 2010	February 2011	February 2011	February 2011	March 2012	March 2012
Treatment	Control	Control	Control	Control	Nitrogen	Control	Control
Number of screened clones	21,755	18,540	10,830	15,105	26,818	25,080	25,080

*Control treatment included ambient precipitation and ambient nitrogen deposition, while the nitrogen treatment had nitrogen enrichment and ambient precipitation*.

Primary libraries were plated on LB agar supplemented with 12.5 μg/ml chloramphenicol, and colonies were picked to 96-well Costar microtiter plates (Corning, Tewksbury, MA, USA) containing LB broth with 10% glycerol and 12.5 μg ml^−1^ chloramphenicol using Qpix2 colony picker (Molecular Devices, Sunnyvale, CA, USA). Ninety-five clones were picked to each plate with one well used as a negative control. Plates were incubated at 37°C for 24 h with shaking at 225 rpm followed by storage at −80°C.

### Optimization of the microtiter plate screening assays

Eleven polymer analogs were selected for screening of carbohydrate hydrolytic activities involved in different stages of cellulose, hemicellulose, starch, chitin, and lignin degradation in liquid culture (Table 2). The substrates were chosen to take advantage of different detection modalities (fluorescence and colorimetric) that could be performed simultaneously, and also to target two to three distinct reactions in the polymer hydrolysis to enable multiplexing within the same assay.

**Table 2:** **Screening substrates**.

**Function**	**Enzyme**	**Substrate**	**Substrate concentration**	**Detection (nm)**	**Assay type**
Cellulose degradation	Cellulase	AZCL-HE-Cellulose	0.1% (w/v)	590	Liquid
	Cellobiohydrolase	4-MUB-β-D-Cellobiose	1 mM	365/450	Liquid
	β-Glucosidase	PNP-β-D-Glucoside	1 mM	410	Liquid
Hemicellulose degradation	Xylanase	AZCL-Xylan	0.1% (w/v)	590	Liquid
	β-Xylosidase	4-MUB-β-D-Xyloside		365/450	Liquid
Starch degradation	α-Amylase	Starch Azure	0.5% (w/v)	590	Liquid
	α-Glucosidase	4-MUB-α-D-Glucoside	1 mM	365/450	Liquid
Chitin degradation	Chitinase	Chitin azure	0.1% (w/v)	590	Liquid
	N-AcetyL-β-D-glucosaminidase	4-MUB-β-N-acetylglucosamine	1 mM	365/450	Liquid
Lignin degradation	Polyphenol oxidase	L-dihydroxyphenylalanine	5 mM	480	Liquid
	Laccase	Syringaldazine	40 μM	530	Liquid
PO_4_ mineralization	Phosphatase	5-Bromo-4-chloro-3-indolyl phosphate	0.1 mM	Visual	Solid
Protein turnover	Protease	Skim milk	2%	Visual	Solid

*In order to allow simultaneous detection of multiple activities, azurin cross-linked (AZCL), p-nitrophenyl (PNP) and 4-methylumbelliferyl (MUB) labeled carbohydrates were pooled on the same assay plate per polymer type, e.g., AZCL Xylan and -MUB- β-D-Xyloside were pooled for screening for activities involved in hemicellulose degradation*.

**Figure 2:**
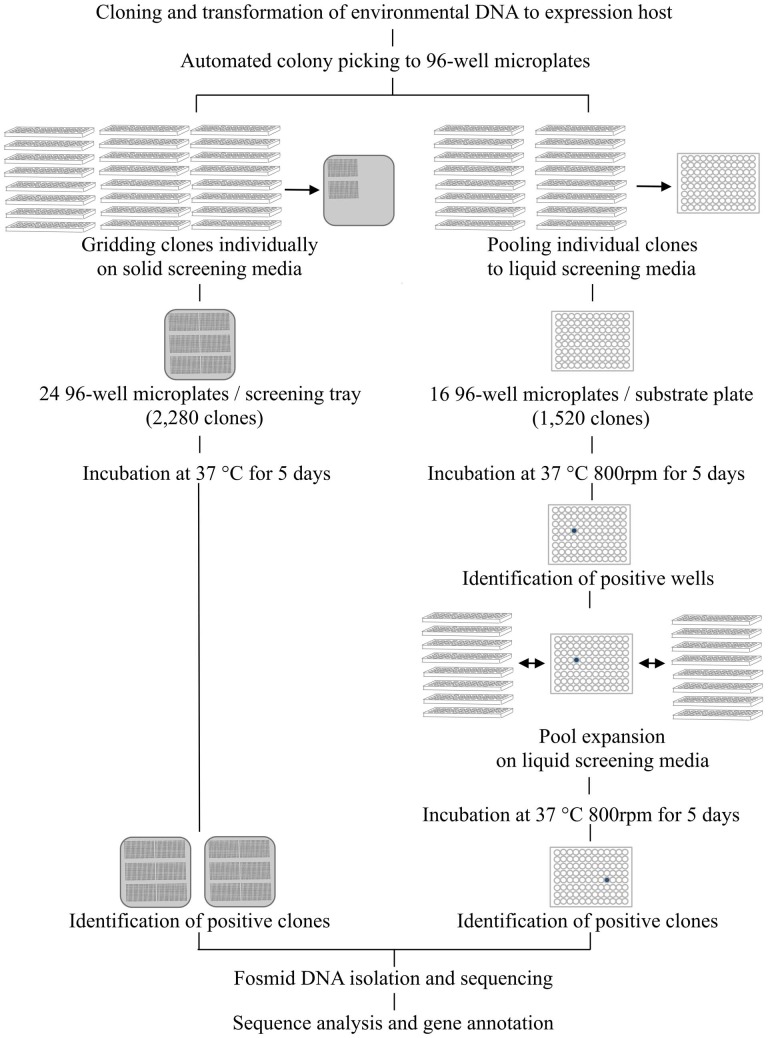
**Screening workflow combining automated colony picking and liquid handling.** In solid media assays clones are individually gridded on agar trays containing screening media. In solution-based assays a two-step clone pooling approach is employed. First, 16 × 96-well plates are combined on one 96-well substrate plate so that each well contains 16 different clones. After identification of positive wells, the 16 clones pooled in the respective well are expanded for individual screening. By repeating the pooling and gridding eight times, 12,160 clones can be screened simultaneously for 14 different activities in a total of 170,240 reactions.

Carbohydrate hydrolytic enzymes and oxidative enzymes were screened on liquid media in two phases. The screening media was LB broth (pH 6 or 6.5) supplemented with 12.5 μ g ml^−1^ chloramphenicol, 1x Fosmid Autoinduction Solution (Epicentre) and screening substrates. Rich growth medium (LB broth) was used to obtain greater quantities of biomass. First, 16 × 96-well microtiter plates obtained from colony picking, containing a total of 1520 clones, were pooled on one 96-deep well assay plate containing screening media (700 μ l in each well) with Biomek FX Workstation (Beckman Coulter, Indianapolis, IN, USA) (Figure 2). Ninety-six-well plates were used instead of 384-well plates to improve detection sensitivity as larger incubation volumes resulted in greater biomass improving the detection of poorly expressed activities. These assay plates were then incubated in the dark at 37°C at 800 rpm in a Microtiterton microplate shaker (ATR Biotech, Laurel, MD, USA). Incubation was continued for 5 days for cell lysis and release of expressed proteins to occur naturally. Absorbance and fluorescence readings were recorded after 24 h and 5 days using an automated workflow on an integrated Biomek FXp Workstation (Beckman Coulter) with Cytomat hotel (Thermo Scientific, Waltham, MA, USA), 6K15 centrifuge (Sigma Laborzentrifugen GmbH, Osterode, Germany), fly-by bar code reader (Beckman Coulter), and Paradigm multimode scanner (Molecular Devices). Bacterial biomass was collected on the bottom of the assay plates by centrifugation at 3000 rpm for 5 min and 100 μ l of supernatant was transferred to a new 96-well microtiter plate for scanning. Wells in which the absorbance or fluorescence signal was greater than a threshold (two standard deviations above the average signal across all wells on the respective plate) were designated positive. This pooling and screening process was repeated eight times to enable processing of 12,160 clones per day. After identifying positive wells from the assay plates from the first phase of screening, individual clones pooled to the positive well were expanded onto new 96-deep well assay plates using the Biomek FXp Workstation and screened individually against the same substrates to identify the clone(s) responsible and to confirm the detected activity (Figure 2).

### Optimization of the solid media screening assays

Solid agar based screening assays for acid and alkaline phosphatases and proteases were established in order to target more general activities in biomass turnover. These assays were combined with automated gridding of individual clones on substrate-containing agar plates to allow direct visual identification of positives without pooling (Figure 2). For phosphatase and protease screening, fosmid clones were gridded on 22 cm × 22 cm bioassay trays (Genetix, New Milton, Hampshire, UK) using a QPix2 colony picker (Molecular Devices) equipped with 96-well gridding head. The screening media was LB agar supplemented with 12.5 μ g ml^−1^ chloramphenicol, 1x Fosmid Autoinduction Solution (Epicentre). Screening substrate was added to the media (Table 2). Acid and alkaline phosphatases were screened for at pH 6 and 8, respectively, and proteases at pH 7. A total of 2280 clones (24 × 96-well plates) were gridded on each bioassay tray. The plates were incubated at 37°C for 5 days and monitored daily for color or halo development.

### Assay validation with purified enzymes and control library

Validation of the microtiter plate screening assays was performed with commercially available purified enzymes (Table S2). Ten-fold dilution series of the enzymes were mixed with screening media (LB broth containing 12.5 μ g ml^−1^ chloramphenicol, 1x Fosmid Autoinduction Solution and screening substrates) on 96-well Costar microtiter plates (Corning) and incubated for 48 h at 37°C in the dark. The plates were scanned with Gemini XPS microplate reader (Molecular Devices) after 6, 24, and 48 h. Limit of detection was determined as the lowest enzyme concentration that was above background signal measured from negative controls.

All screening assays developed were specific for the target activities. The tested cellulase and xylanase enzymes were also active on the respective homopolymers, cellobiose, and xyloside. In most substrates with the exception of 4-MUB-α-D-Glucoside, 4-MUB-β-D-Xyloside and 4-MUB-β-N-acetylglucosamine, no background activity was detected. In these three substrates, the background activity corresponded to 1 × 10^−3^–100 U ml^−1^ of purified enzyme (Table S2).

Non-specific background arising from metabolic activity of the expression host was measured by incubating the control library under the assay conditions and recording fluorescence and absorbance readings. Abiotic hydrolysis of substrates was confirmed to be negligible within the time frame of the assays (data not shown).

### Sequencing of fosmid clones

Fosmid DNA was isolated from copy number induced overnight cultures using a 96-well plasmid miniprep procedure (Whatman, Clifton, NJ, USA) and Biomek FX Workstation. Cells were lysed using alkaline lysis and passed through Lysate Clarification plate (Whatman). DNA was bound on a DNA Binding Plate (Whatman), washed and then eluted with 70°C Tris buffer. Fosmid DNA was then run on 1% agarose gel in 0.5 × TAE buffer at 100 V for 4 h to separate residual genomic DNA. Gel fragments containing purified fosmid DNA were excised from the agarose gel and mixed with three volumes of gel extraction buffer [6 M Guanidine thiocyanate, 50 mM Tris-HCl (pH 7.5), 20 mM EDTA (pH 8.0)]. The mixture was heated to 50°C for 5 min, mixed with one gel volume of isopropanol and the mixture was passed through the DNA Binding Plate (Whatman). Fosmid DNA was eluted with 70°C 10 mM Tris buffer and quantified using the Quant-iT PicoGreen dsDNA Reagent (Invitrogen) with detection using a Gemini XPS microplate reader.

In order to determine the phylogenetic diversity of DNA captured in the libraries, a subset of clones was randomly selected from each library and subjected to end-sequencing using Sanger sequencing at the UC Berkeley DNA Sequencing Facility (Berkeley, CA, USA).

For shotgun sequencing of insert DNA from a subset of active clones, 95 fosmid extracts were individually dual indexed using the Nextera XT DNA Sample Preparation and Nextera Index kits (Illumina Inc., San Diego, CA, USA) according to manufacturer's protocol. PhiX was used as a library preparation and sequencing control. The individually indexed sequencing libraries were normalized and pooled according to the Nextera XT DNA Sample Preparation kit recommendations and sequenced from both ends with the 500-cycle MiSeq Reagent Kit v2 on MiSeq (Illumina Inc.).

### Sequence analysis of fosmid clones

End-sequences derived from Sanger sequencing were base called with Phred (Ewing and Green, 1998; Ewing et al., 1998) and compared to the NCBI non-redundant protein sequence database (nr) using Blastx (Altschul et al., 1990). The phylogenetic composition of libraries was then inferred from the Blastx output using MEGAN4 (Huson et al., 2011).

Sequence reads obtained from MiSeq were base called and demultiplexed on BaseSpace (Illumina). The demultiplexed reads were quality trimmed using trimmomatic with the following parameters: headcrop 11, trailing 20, sliding window 4:20, minimum length 75 bp (Lohse et al., 2012). Reads matching to the cloning vector and *E. coli* genome were removed following mapping with Bowtie (Langmead et al., 2009). The trimmed and filtered sequences were then assembled with Velvet (version 1.2.07, Zerbino and Birney, 2008) using the following parameters: k-mer size: 65, expected coverage: estimated, coverage cut-off: 4, insert length 1000 bp. Assembled scaffolds were subjected to gene prediction using Prodigal that was run in metagenomic mode (Hyatt et al., 2010). Amino acid sequences of predicted open reading frames were then searched against the database for automated carbohydrate-active enzyme annotation (dbCAN, Yin et al., 2012), the Laccase and Multicopper Oxidase Engineering Database (LAMOED, Sirim et al., 2011), the Peroxidases Database (PeroxiBase, Fawal et al., 2013), and the Pfam 26.0 database (Punta et al., 2011) using HMMER (version 1.0.0) and workflows employed in Galaxy (Giardine et al., 2005; Blankenberg et al., 2010; Goecks et al., 2010). *E*-value threshold for annotations was set to 1 × 10^−1^. If several annotations were obtained for an ORF, annotation with the lowest *e*-value was selected for further analyses. In order to compare *in silico* annotations to biochemically confirmed activity, the annotated glycoside hydrolase (GH) genes were related to substrates using known activities reported for the respective GH families in the Carbohydrate-active enzymes database (Figure S2) (Cantarel et al., 2009).

Fosmids were assigned to genome bins based on their TNF patterns following a methodology similar to the one outlined in Dick et al. (2009). Only sequences from clones with confirmed biochemical activity and that were at least 2 kb long were included in the analysis. All sequences were fragmented into 2 kb non-overlapping fragments (subsequences). TNFs were calculated for each fragment using a custom R script (R Core Team, 2012) and the resulting data matrix was used to construct a self-organizing map (SOM) using Databionics ESOM tools (Ultsch and Moerchen, 2005). The map size was adjusted according to the number of sequence fragments for training (5.5 neurons per data points) and the starting value for training radius was set to 24. Clusters (genome bins) of sequence fragments were identified following a visual inspection of the map. Each sequence was assigned to the genome bin that included the majority of its fragments. Sequence fragmentation and downstream processing were done using in-house custom R scripts (R Core Team, 2012).

Phylogenetic marker genes (*gyr*B, *rec*A, transcription elongation factors and ribosomal proteins) for taxonomic assignment of the TNF bins were identified from Pfam output. The corresponding ORFs were searched against the nr using Blastp (Altschul et al., 1990) and ten most significant hits were retrieved for sequence alignment. The amino acid sequences were aligned with ClustalX (Larkin et al., 2007) and manually edited in Seaview (Gouy et al., 2010). Best-fit model for amino acid substitution was selected with Prottest3 (Darriba et al., 2011) and maximum-likelihood trees were constructed with PhyML 3.0 (Guindon et al., 2010) using the substitution model selected in Prottest3 and 100 permutations. GH10 endo-β-1,4-xylanase sequences were analyzed in a similar manner except that the tree was visualized using iTOL (Letunic and Bork, 2006, 2011).

### Sequence accession numbers

Fosmid sequences and annotations have been deposited to GenBank under accession numbers KF524439-KF524837.

## Results

### Screening of leaf litter metagenomic libraries

In this study, we developed automated functional screening assays for function-based characterization of activities related to plant polymer decomposition contained in environmental metagenomes. The fully automated screening assays employ a clone pooling and substrate multiplexing strategy coupled with multimodal detection, and enable simultaneous screening of 12,160 individual clones for 14 distinct activities. The 170,240 individual screening assays can be prepared in a day, followed by a 5-day incubation and expansion of positive clones.

To demonstrate the applicability of the screening platform in high throughput screening of metagenomic libraries, we prepared seven fosmid libraries from decomposing leaf litter and screened 143,228 clones in a total of 2,005,192 screening assays (Table 1). This screening resulted in 444 positive assays with the frequency of positive assays ranging from 0.003% for more complex carbohydrates such as AZCL HE-Cellulose to 0.09% for 4-MUB-β-D-Xyloside (Table 3). No activity was detected in chitinase, laccase, or polyphenol oxidase assays. Fifty-nine clones showed activity on two or three different screening substrates resulting in 374 unique positive clones and an average positive rate of 0.26% (Table 3).

**Table 3:** **Number of positive assays observed during the screening of leaf litter metagenomic libraries with the functional screening platform developed in this study**.

**Library**	**Dec10_01XX**	**Dec10_08XX**	**Feb11_01XX**	**Feb11_08XX**	**Feb11_16XN**	**Mar12_01XX**	**Mar12_08XX**
**Number of clones**	**21,755**	**18,540**	**10,830**	**15,105**	**26,818**	**25,080**	**25,080**
AZCL-HE-cellulose	–	1	–	–	4	–	–
4-MUB-β-D-cellobiose	12	23	13	9	31	6	6
PNP-β-D-glucopyranoside	6	11	9	6	14	4	1
AZCL-Xylan	5	5	7	6	6	2	1
4-MUB-β-D-Xyloside	24	19	20	11	31	14	8
Starch azure	2	1	2	–	1	1	–
4-MUB-α-D-glucoside	4	4	–	2	3	–	–
Chitin azure	–	–	–	–	–	–	–
4-MUB-β-N-acetylglucosamine	8	16	7	15	19	2	4
L-dihydroxyphenylalanine	–	–	–	–	–	–	–
Syringaldazine	–	–	–	–	–	–	–
5-Bromo-4-chloro-3-indolyl phosphate	9	10	4	3	7	–	1
Skim milk	–	1	1	–	–	–	2

### Sequence analysis of positive clones

**Figure 3:**
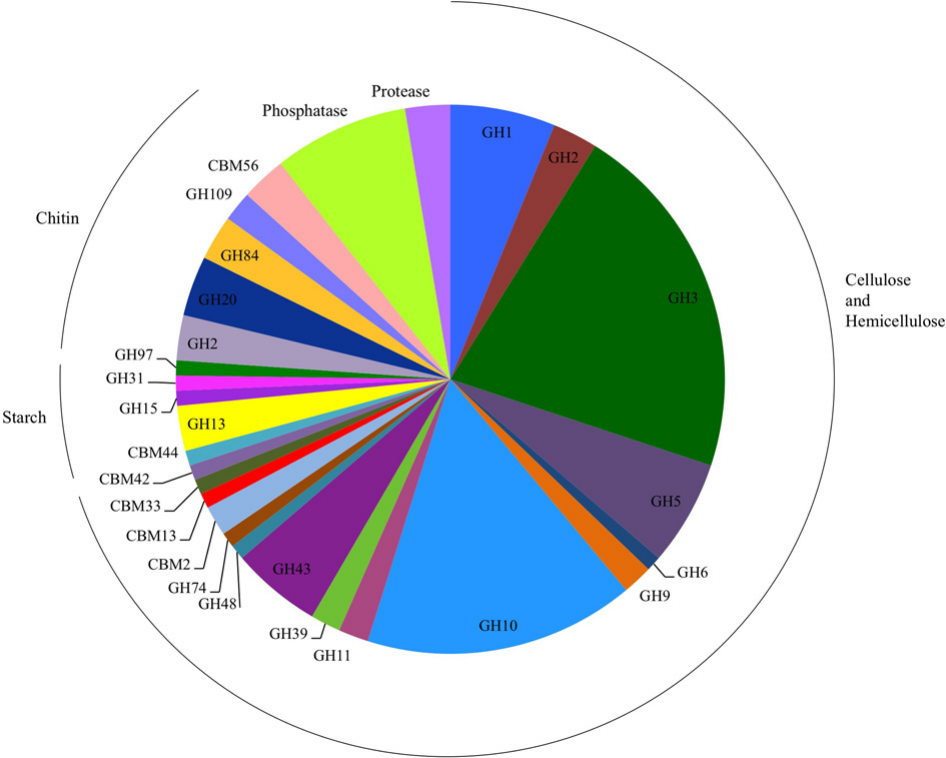
**Number of glycosyl hydrolase (GH) families, GH gene-associated carbohydrate binding modules (CBM), phosphatases, and proteases detected in the sequenced clones.** Clones detected on chitin substrates were active only on 4-MUB-β-N-acetylglucosamine.

To identify the genetic basis for the detected activities, we selected 95 clones with biochemical activity from the seven libraries for insert DNA sequencing using the Illumina MiSeq platform (Table S3). The average size of the total assembly per clone was 35,537 (±5873) base pairs and consisted of one to eight scaffolds. Gene prediction showed 33 (±8) genes per fosmid, and genes corresponding to the biochemically confirmed activity were successfully identified from 79 of the 95 sequenced clones following HMM searches using profiles derived from the dbCAN and Pfam databases (Punta et al., 2011; Yin et al., 2012). The highest diversity of GH families, a total of 12 distinct families, was detected in cellulose- and hemicellulose-hydrolyzing clones, among which GH families 3 and 10 were the most abundant (Figure 3). In starch-hydrolyzing clones and clones active on 4-MUB-β-N-acetylglucosamine, the identified carbohydrate-active genes represented GH families 13, 15, 31, and 97 and GH families 2, 20, 84, and 109, respectively. In two cellulose- and hemicellulose-hydrolyzing clones only carbohydrate binding modules (CBM) were detected.

**Figure 4:**
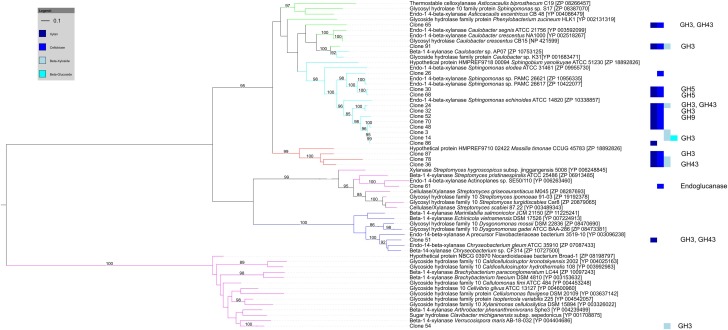
**Maximum likelihood tree of GH10 endo-β-1,4-xylanases identified from cellulose and hemicellulose hydrolyzing fosmid clones.** Clone numbers indicate clones from which the GH10 genes were identified and refer to Table S3. The screening substrates that the clones were active on are indicated by bar colors (Dark blue: AZCL Xylan; medium blue: MUB-β-D-Cellobiose; cyan: 4-MUB-β-D-Xyloside; Light blue: PNP-β-D-glucopyranoside). Additional GH genes identified by homology-based annotation from the respective clones are shown on the right. Branches are colored by tetranucleotide frequency bin (Green and cyan: Bins 7 and 10, α-proteobacteria; Red: Bin 8, β-proteobacteria; Blue: Bin 5, Bacteroidetes; Magenta: Bin 1, Actinobacteria). Bootstrap percentages from 100 permutations higher than 80% are shown at branches. The scale bar represents the number of amino acid substitutions.

Of the 27 cellulose and hemicellulose hydrolyzing clones that displayed activity on more than one screening substrate, ten clones had more than one carbohydrate-active gene. In all ten clones, the predicted activities based on the gene content agreed with the biochemically-confirmed activities. In the remaining 17 clones that showed activity on multiple substrates, only one carbohydrate-active gene was identified bioinformatically. These comprised GH1 or GH3β-glucosidases that were active on 4-MUB-β-D-Cellobiose, 4-MUB-β-D-Xyloside and/or PNP-β-D-glucopyranoside, GH10 endo-β-1,4-xylanases active on both AZCL Xylan and 4-MUB-β-D-Cellobiose (Figure 4), or a GH9 cellulase active on AZCL-HE-Cellulose and 4-MUB-β-D-Cellobiose. Three clones were identified to carry GH10 endo-β-1,4-xylanases but showed no activity on AZCL Xylan (Figure 4).

The 16 clones in which we were not able to identify carbohydrate-active enzymes or CBMs were screened additionally to re-assess their activity. In ten clones in which the absorbance or fluorescence signal was originally close to our defined positive threshold, the activities could not be reliably reproduced. In the remaining six clones, the observed activity was reproduced despite the absence of any clear gene homologs.

### Phylogenetic diversity of the positive clones

**Figure 5:**
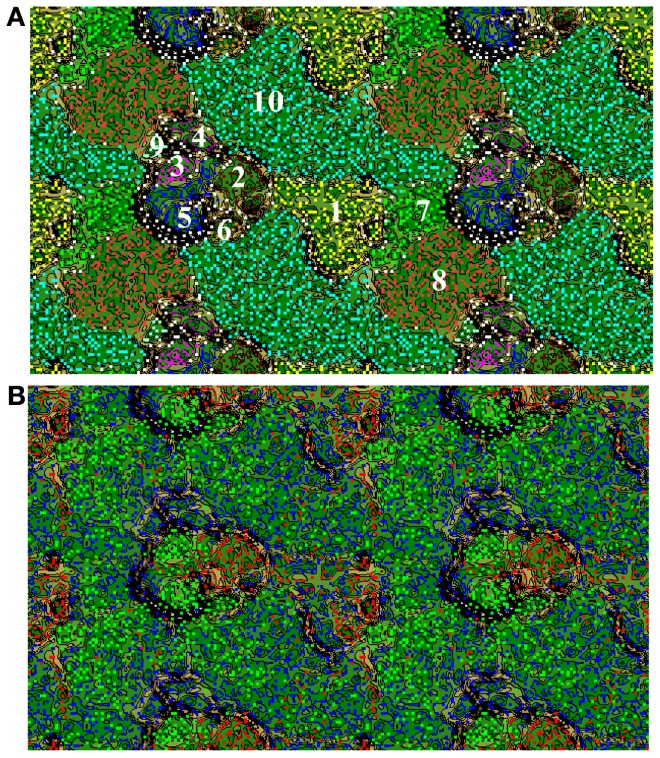
**(A)** Emergent self-organizing map (ESOM) of the sequences from the positive clones. The map was generated based on tetranucleotide frequencies calculated from contigs fragmented to 2 Kbp. In ESOM, sequences with similar tetranucleotide frequencies cluster together in multidimensional space. Here, these clusters, i.e., bins, are indicated with numbers with phylogenetic affiliations as follows: (1) Actinobacteria, (2) Fungi, (3) Bacteroidetes, (4) Bacteroidetes, (5) Bacteroidetes, (6) γ-Proteobacterial, (7) α-Proteobacteria, (8) β-Proteobacteria, (9) γ-Proteobacteria, and (10) α-Proteobacteria. **(B)** ESOM with sequence fragments colored by the sampling time point that leaf litter was collected for fosmid library construction (Green: December 2010; Blue: February 2011; Red: March 2012).

For the 79 clones with confirmed biochemical activity, we used TNF binning to link the identified functional traits to phylogenetic units. Based on their TNF patterns, the analyzed clones clustered into ten TNF bins (Figures 5A,B). The three most prominent bins (7, 8, 10) contained 71.2% of the clones (57 clones). Based on the presence of conserved phylogenetic marker genes, the three bins comprised DNA from α- and β-Proteobacteria (Figure S3). The α-Proteobacterial bins 7 and 10 included cloned DNA with database homology to proteins from the *Caulobacteraceae*, *Sphingomonadaceae*, and *Rhizobiaceae* families. The β-Proteobacterial bin 8 contained DNA homologous to proteins from the *Oxalobacteraceae* and *Comamonadaceae* families. The other two phylogenetically assigned bins included γ-Proteobacterial (bin 6, *Xanthomonadaceae*) and Bacteroidetes (bin 5, *Sphingobacteriaceae*) clones. The remaining bins were not phylogenetically assigned due to the lack of conserved marker genes. According to similarity searches of ORFs against the nr database these bins consisted of clones originating from Actinobacteria (bin 1), Fungi (bin 2), Bacteroidetes (bin 3 and 4), and γ-Proteobacteria (bin 9).

**Figure 6:**
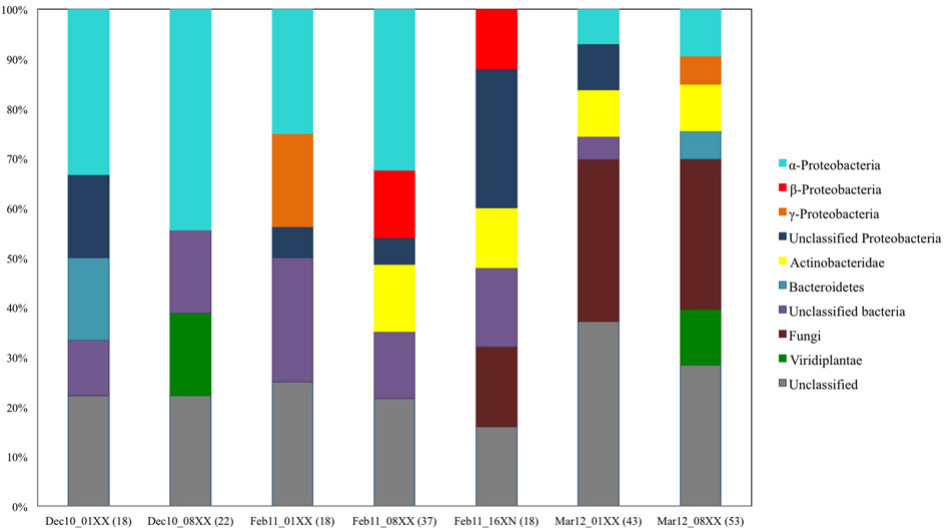
**Summary of phylogenetic composition of the seven leaf litter fosmid libraries constructed and screened in this study.** The phylogenetic origin of the insert DNA was determined by blastx search of the insert DNA end-sequence against the non-redundant protein sequence database of NCBI.

In order to determine the degree of phylogenetic diversity captured by our screening approach we compared the observed diversity among the sequenced clones with the diversity of the libraries used for screening (Figure 6). In the clones with biochemically-confirmed activity originating from the December 2010 and February 2011 leaf litter libraries, almost 48% of clones were grouped to Proteobacterial bins and nearly 8% to Actinobacteria. In the respective complete libraries (all clones), the abundance of these phylogenetic groups was 40–51% and 0–13.5%, respectively (Figures 5B, 6). In the March 2012 libraries, almost 47% of biochemically-confirmed clones were assigned to Actinobacteria compared to 9% in the complete library while the relative abundance of Proteobacterial clones was 40% in the biochemically confirmed clones compared to almost 16% in the complete library (Figures 5B, 6).

### Functional trait linkage

**Figure 7:**
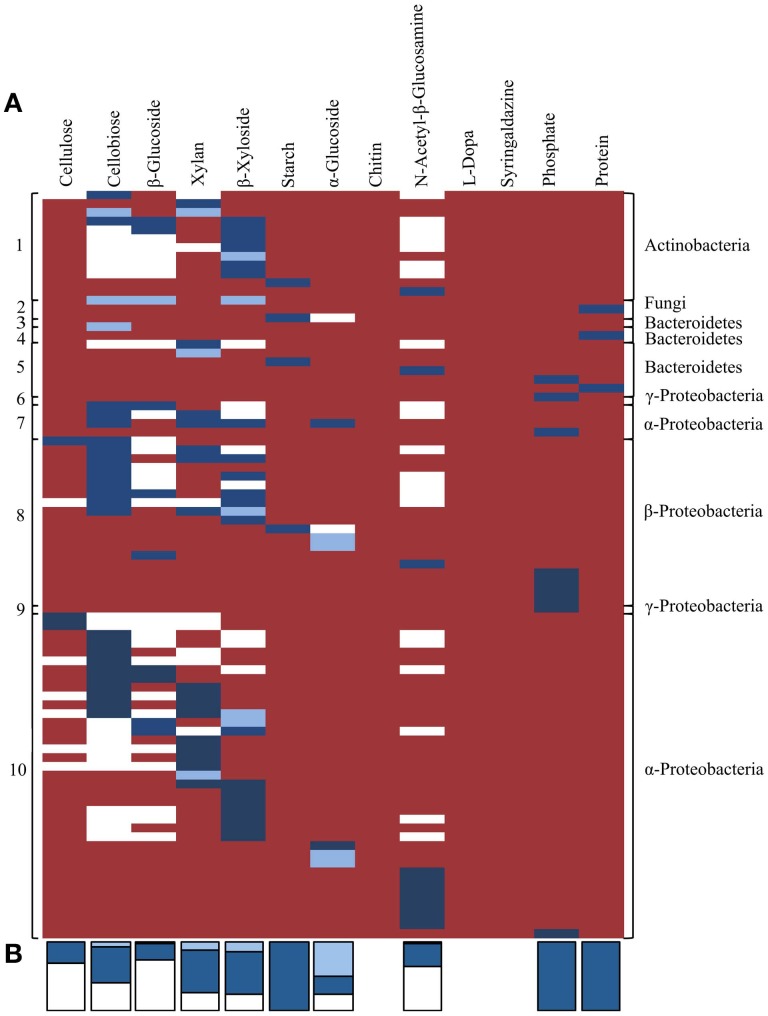
**(A)** Co-occurrence of traits related to plant polymer decomposition within tetranucleotide frequency bins, derived by either *in silico* predicted and/or activity-based screens. Each row represents an individual sequenced fosmid with biochemically confirmed activity against one or more substrates. White shading indicates activities predicted by *in silico* annotation only. Light blue shading indicates activities that were biochemically confirmed using functional screening but not predicted by *in silico* annotation. Dark blue shading indicates activities predicted by *in silico* annotation and biochemically-confirmed by functional screening. Red shading indicates no predicted or observed activity. Numbers on the left correspond to TNF bins in Figure 5. **(B)** Stacked bars at the bottom indicate the proportion of activities for each substrate predicted by homology-based *in silico* annotation and/or functional screening. Colors correspond to panel **(A)**.

Lastly, we compared distribution of the traits identified by activity-based screening and homology-based functional annotation within the TNF bins in order to investigate potential phylogenetic distribution of the detected traits and their linkage within taxonomic groups. In general, when considering the presence of a function by gene annotation and/or biochemical activity most TNF bins possessed most of the assayed activities (Figure 7). According to homology-based *in silico* functional annotation, activities involved in cellulose and hemicellulose hydrolysis were evenly distributed across all detected microbial taxa. The *in silico* functional annotations also suggested that the predicted carbohydrate-active genes were active on a higher number of substrates than was biochemically confirmed. Biochemically confirmed cellulolytic activities were most often encountered in the α- and β-Proteobacterial bins where 44% of the biochemically confirmed activities involved cellulose, cellobiose and β-glucoside hydrolysis. In the Actinobacterial bin, 58% of the clones were active on 4-MUB-β-D-Xyloside. The protease positive clones were detected only in fungal and Bacteroidetes bins.

### Homology-based and biochemically confirmed functional predictions

The biochemically confirmed activities on Starch Azure, phosphatase, and skim milk were fully concordant with activities predicted by homology-based annotations (Dark blue bars in Figure 7). However, biochemical confirmation of the annotated activity was only observed in 70% of the predicted hemicellulose hydrolytic activities, approximately 50% of the predicted cellobiose and α-glucoside hydrolytic activities and in less than 33% of predicted activities on cellulose, β-glucoside, and N-acetyl-β-glucosamine (Dark blue bars in Figure 7). In 6 clones with biochemically confirmed activity no genes with appropriate predicted activities were detected and in 3 clones activity was observed on substrates not predicted by *in silico* annotation (Light blue bars in Figure 7).

## Discussion

Functional metagenomics has great potential in providing direct linkages between genes, their activity, and their phylogenetic context and ecological role. However, shortcomings in screening throughput and sensitivity currently limit the wider exploitation of function-driven mining of metagenomic libraries in complementing environmental sequencing efforts. In this study, we developed a method for high throughput screening of activities involved in cellulose, hemicellulose, chitin, starch and lignin decomposition, organic phosphate mineralization and protein hydrolysis from environmental metagenomes.

We used several strategies to address the shortcomings in analysis throughput, sensitivity and specificity. Firstly, we used multiple substrates with different detection modalities (fluorescence and colorimetric) to increase screening throughput. In addition to enabling direct screening of targeted activities without further staining steps, this multiplexing of labeled substrates allowed simultaneous detection of different activities required during different stages of biomass depolymerization. Secondly, by combining the multiplexed screening approach with a two-step clone pooling strategy and automated liquid handling we were able to further increase the number of screened clones 16 times as well as to further reduce the cost of screening. Thirdly, by genetically modifying the expression host to reduce wild-type amylase and phosphatase activities that can interfere with assay sensitivity and specificity we improved signal-to-noise ratio and sensitivity of the starch and phosphatase assays. We also included fosmid copy number induction in all screening assays because higher vector copy number can be advantageous for identification of genes that were cloned without promoters or have otherwise low expression efficiency (Martinez et al., 2007).

Using the developed screening assays we identified carbohydrate hydrolytic activities, phosphatases, or proteases in seven fosmid libraries prepared from decomposing leaf litter. The highest frequency of positive clones was found on small oligosaccharide substrates and 5-bromo-4-chloro-3-indolyl phosphate, which is in agreement with the high frequency (32–79%) of these activities in the sequenced bacterial genomes (Berlemont and Martiny, 2013; Zimmerman et al., 2013). According to shotgun sequencing of a subset of the active clones identified by screening and homology-based gene annotation the carbohydrate-active genes represented a range of different GH families. The highest diversity in the identified GH families was detected in cellulose and hemicellulose hydrolyzing clones. In accordance with the biochemically confirmed activities the most abundant GHs identified in the sequenced clones included small oligosaccharide processing GH3 β-glucosidases and β-xylosidases. This further supports conclusions from their frequency in sequenced bacterial genomes (79% of genomes have at least one; Berlemont and Martiny, 2013).

The clones with confirmed biochemical activity represented several distinct sub-phyla, α-, β- and γ-Proteobacteria, Sphingobacteria (Bacteroidetes), and Actinobacteria, as well as Fungi, each phylogenetically distinct from the *E. coli* expression host. The detected phyla corresponded well with the taxonomic diversity of the screened libraries. Together these results demonstrated that activity-based mining of metagenomic libraries can effectively capture functional and taxonomic diversity present in environmental microbial communities.

In most cases the biochemically confirmed activities were concordant with activities predicted by homology-based annotations and the carbohydrate hydrolytic activities corresponded to the reported substrate ranges of the detected GH families (Biely et al., 1997; MacGregor et al., 2001; Cournoyer and Faure, 2003; Hill and Reilly, 2008; Slámová et al., 2010a,b; Berlemont and Martiny, 2013). This showed that in some cases *in silico* predictions for functional annotation may be reliably used. However, the predictive ability of homology-based annotation was lower for the hydrolytic activities involved in cellulose, hemicellulose, chitin and alpha-glucoside decomposition. In this case, functional screening enabled some constraints to be placed on the broad substrate ranges that would be predicted by homology-based annotation, further demonstrating the potential of activity-based screening in providing new enzyme substrate linkages and constraining activities predicted by *in silico* annotations.

The activity-based screening enabled linkage of biochemically confirmed functions on two levels. As demonstrated by the detection of clones displaying multiple substrate activities, the concurrent use of different screening substrates and large insert fosmid metagenomic libraries provided data about the co-occurrence of these activities within the same genomic regions. Binning of the fosmid sequences based on TNF patterns enabled further association of functions to genomes or closely related genomes with defined phylogeny. Overall, biochemically confirmed activities involved in processing of small oligosaccharides, such as β-glucosidases, β-xylosidases, and β-N-acetylhexosaminidases, were found across all of the detected bacterial phyla; Proteobacteria, Sphingobacteria (Bacteroidetes), and Actinobacteria. This is concordant with recent reports showing that these activities are found across nearly all bacterial phyla in sequenced genomes (Berlemont and Martiny, 2013; Zimmerman et al., 2013). However, in contrast to sequenced microbial genomes in which cellulolytic activities were observed to be present in both Proteobacteria and Actinobacteria (Berlemont and Martiny, 2013), the biochemically confirmed cellulolytic activities were most often encountered in the α- and β-Proteobacterial bins. Fosmids derived from Actinobacteria were predominantly active on 4-MUB-β-D-Xyloside. This observation may result from local selective pressure in the ecosystem of study. It may also indicate that sequenced representatives in public databases are not representative of the functional potential of these microbial communities, or that actinobacterial cellulolytic enzymes are not active on the substrates used in this study.

Although differences in tetranucleotide usage patterns can be used as signatures to assign genomic fragments to microbial species or closely related taxa (Teeling et al., 2004; Dick et al., 2009), incorrect assignment of heterologous clones can be a concern. Also in some cases, the phylogenetic assignment of genomic fragments within TNF bins can be limited by the occurrence of phylogenetically informative markers. For example, with the sequence data available it was not possible to classify many of the TNF bins at finer phylogenetic resolution. Nevertheless, we anticipate that our ability to further refine bin classification and phylogenetic assignment will improve with the incorporation of sequence data from additional active clones and by incorporation of assemblies derived from non-targeted metagenomic sequence surveys.

Together, activity-based screening allowed substrate specificities to be constrained for the identified enzymes and provided further empirical support for substrate specificity of functions predicted *in silico*. Although our data demonstrated that in some cases using *in silico* predictions to infer activity may be reliable, relying solely on *in silico* predictions for all functions of interest can be misleading. For instance, we successfully reproduced activity in six clones but we could not identify any genes corresponding to the detected activity—i.e., *in silico* false negatives. We also detected three clones with GH10 endo-β-1,4-xylanases that showed no activity on AZCL Xylan, but were, instead, detected on other substrates as a result of expression of other enzymes from the same fosmids—i.e., *in silico* false positives. We cannot rule out the possibility that these enzymes may not display activity against the specific substrates used in our study. This is a common limitation of all activity screens using non-natural substrates chosen for their colorimetric or fluorogenic properties. Further experimental work to define the substrate ranges of *in silico* predicted enzymes at a scale that matches the screening approach described here would be highly complementary. Although we typically use expression of any protein on a fosmid as an indicator of expression host compatibility, it is possible that enzymes, e.g., GH10 enzymes in this study, were not expressed in their fully functional form in the *E. coli* host. Differences in transcription, expression, and post-transcriptional processing mechanisms between the host and the taxonomically diverse organisms could lead to this (Gabor et al., 2004; Park et al., 2007). Such constraints related to protein expression and substrate specificity may also explain the absence of lignin and chitin degrading activities as well as the limited detection of protease activities in this study. To alleviate limitations in expression, extended host-range shuttle vectors (Aakvik et al., 2009) and alternative expression hosts (Craig et al., 2010; Kellner et al., 2011) could be employed. However, in another study of gut microbiota from lignocellulose degrading insects we have observed lignin degrading activities using the same protocols employed here (data not shown), suggesting that expression of these proteins is not the primary limitation. Nevertheless it is clear from the data presented here, that the prediction of multiple enzyme-substrate relationships based on *in silico* prediction alone is likely to result in inaccurate predictions of the functional roles of organism in such environments. Therefore a complementary approach of functional screening combined with metagenomic prediction is recommended.

## Conclusions

Activity-based screening of metagenomic libraries has great potential to bridge the gap between sequence data generation and functional predictions, while also providing a path for assigning and linking functional traits at a genomic level within microorganisms. However, in order to be ecologically relevant, functional screening must be capable of interrogating the potential activity of microbial communities, in a manner that is both deep, i.e., covering lower abundance organisms, and broad in assessing the potential range of activities of those organisms. Recent advances in sequencing technologies are delivering new predictions and hypotheses about the functional roles of environmental microorganisms, yet until we can test these predictions at a scale that matches our ability to generate them, most will remain as hypotheses. This study represents an attempt to begin this scaling of biochemical confirmation, and can be improved and built upon. We have developed automated screening assays for function-based mining of a subset of activities important to ecosystem functioning. These assays not only enable identification of novel hydrolytic and oxidative enzymes but can also provide insight into substrate specificity of these enzymes, their co-occurrence patterns with other functions, and their distributions across microbial populations. By using this information in tandem with metagenomic surveys and environmental genome reconstruction, we can begin to build a more complete picture of microbial functioning and the consequences for ecosystem services of alterations in microbial composition driven by land use and global change.

### Conflict of interest statement

The authors declare that the research was conducted in the absence of any commercial or financial relationships that could be construed as a potential conflict of interest.
