# Supplementary material for: Coupled high-throughput functional screening and next generation sequencing for identification of plant polymer decomposing enzymes in metagenomic libraries
Source: Front Microbiol. 2013 Sep 23;4:282. doi: 10.3389/fmicb.2013.00282 (PMC3779933; doi:10.3389/fmicb.2013.00282)
Supplement: Supplementary file 1 [file DataSheet1.DOCX]

**Supporting information**

**Table S1.** N-terminal and C-terminal deletion primers, locus-specific primers and test primers used for *E. coli* gene disruption.

| **Target gene** | **Protein** | **Primer ID** | **Primer sequence 5’ – 3’** | **Reference** |
| --- | --- | --- | --- | --- |
| **N-terminal and C-terminal deletion primers** | | |  |  |
| *amy*A | Cytoplasmic α-amylase | AmyA_pKD13_Fwd | CCGATTACGGCTACGCTTCTAATGTTCCCCTTGAA  TGGAGTCGAAGAATGATTCCGGGGATCCGTCGACC | Baba et al. (2006) |
|  |  | AmyA_pKD13_Rev | ATCTCCCCGGCTTGCCGCCGGGGATGAATTTAAAT  CACCTCTTCGATAACTGTAGGCTGGAGCTGCTTCG | Baba et al. (2006) |
| *mal*S | Periplasmic α-amylase | malS_pKD13_Fwd | AGGTACTGACGAACGGGAAGCCGGAAAAGTTACTGTTGCCCTGCCCAGACATTCCGGGGATCCGTCGACC | Baba et al. (2006) |
|  |  | malS_pKD13_Rev | ATCGCTGGCAAACTGTCTGAAATCGCAGCAATAAGGACTCATCCGCCATGTGTAGGCTGGAGCTGCTTCG | Baba et al. (2006) |
| *mal*Z | Maltodextrin glucosidase | malZ_pKD13_Fwd | TGCATTAGGCTATGGCAAGGTGATCAGATTTTCATCACAGGGGAATTATGATTCCGGGGATCCGTCGACC | Baba et al. (2006) |
|  |  | malZ_pKD13_Rev | GCAGGCGTCACGCAAGGCGTTATAAAACGTTAGTTCATCCATACCGTAGCTGTAGGCTGGAGCTGCTTCG | Baba et al. (2006) |
| yihQ | α-glucosidase | yihQ_pKD13_Fwd | ATAACAGGAAGGCAACAATCTCGTTCTCAACTCTATGGAGATTAGTTATGATTCCGGGGATCCGTCGACC | Baba et al. (2006) |
|  |  | yihQ_pKD13_Rev | CCCATGAGTTTCCCCACGGGCGGATGTGATTAGATGCTTTTTAACGACGCTGTAGGCTGGAGCTGCTTCG | Baba et al. (2006) |
| *pho*A | Alkaline phosphatase | phoA_pKD13_Fwd | TCGCTTTGTTTTTATTTTTTAATGTATTTGTACATGGAGAAAATAAAGTGATTCCGGGGATCCGTCGACC | Baba et al. (2006) |
|  |  | phoA_pKD13_Rev | GCAGCGAAAATTCACTGCCGGGCGCGGTTTTATTTCAGCCCCAGAGCGGCTGTAGGCTGGAGCTGCTTCG | Baba et al. (2006) |
| *app*A | phosphoanhydride phosphohydrolase | appA_pKD13_Fwd | GCATCAGGCAATCAATAATGTCAGATATGAAAAGCGGAAACATATCGATGATTCCGGGGATCCGTCGACC | Baba et al. (2006) |
|  |  | appA_pKD13_Rev | TCAGGTAACTGAATGCTCTTTTTTATGCATTACAAACTGCACGCCGGTATTGTAGGCTGGAGCTGCTTCG | Baba et al. (2006) |
| *aph*A | Acid phosphatase | aphA_pKD13_Fwd | AATCTCACAATAAAAAGTTTCAACATACTGACTATTTAGGGAAAAATATGATTCCGGGGATCCGTCGACC | Baba et al. (2006) |
|  |  | aphA_pKD13_Rev | TCGGGAGAGCATCACGCTCTCCCGCTCTGTCAGTA  TTCTGAATTGACGATTGTAGGCTGGAGCTGCTTCG | Baba et al. (2006) |
| **Locus-specific primers** | | |  |  |
| *amy*A | Cytoplasmic α-amylase | fliD_Fwd | CCA AAT ACA CCG CCG TAG AT | This study |
|  |  | yedD_Rev | CGTGACGGTAACACCATTGA | This study |
| *mal*S | Periplasmic α-amylase | bax_Fwd | GTGCGTCTTCGCTAACACCT | This study |
|  |  | avtA_Rev | GACCGTCGTAGTTACACAGT | This study |
| *mal*Z | Maltodextrin glucosidase | proY_Fwd | CGTGGATTGTTGTGCTGTTG | This study |
|  |  | acpH_Rev | ATTTAGCCCATCTCGCGGAA | This study |
| *yih*Q | α-glucosidase | yihR_Fwd | TAT GAG TTG CCC GCC TAA CG | This study |
|  |  | yihP_Rev | AGT TGC TGG ATC TTC CGT TG | This study |
| *pho*A | Alkaline phosphatase | iraP_Fwd | GCATTCCTGACGACGATACG | This study |
|  |  | psiF_Rev | GGTTAAAGTTCTCTCGGCAGC | This study |
| *app*A | phosphoanhydride phosphohydrolase | appB_Fwd | GTTGCTCTACACTCTCTGG | This study |
|  |  | etk_Rev | CAGCACCGCTTACAGTTACG | This study |
| *aph*A | Acid phosphatase | tyrB_Fwd | GCA AAT GTA CAA CGT GTG GCA | This study |
|  |  | yjbQ_Rev | CAG ATG CAG TAA GCC GAT GT | This study |
| **Test primers** | |  |  |  |
|  |  | k1 | CAGTCATAGCCGAATAGCCT | Datsenko and Wanner (2000) |
|  |  | k2 | CGGTGCCCTGAATGAACTGC | Datsenko and Wanner (2000) |

**Table S2.** Validation of screening assays with purified enzymes. All assays were performed in Luria Bertani broth at pH 7 and 37 °C. pH and °C show the temperature optima for the purified enzymes as reported by the supplier.

| **Substrate** | **Enzyme** | **Organism** | **Supplier** | **pH** | **°C** | **Lowest amount detected** | |
| --- | --- | --- | --- | --- | --- | --- | --- |
|  |  |  |  |  |  | **ng ml^-1^** | **U ml^-1^** |
| AZCL-HE-Cellulose | Cellulase | *Trichoderma reesei* ATCC 26921 | Sigma | 5 | 37 | <67 | <0.4 |
| 4-MUB-β-D-Cellobiose | Cellobiohydrolase 48A | *Clostridium thermocellum* | Nzytech | 6 | 60 | 500 | n.a. |
| 4-Nitrophenyl-β-D-Glucopyranoside | β-glucosidase | *Rhizobium etli* CFN 42 | Prozomix | 5.4 | 40 | 5 | 10^-4^ |
| AZCL Xylan | Xylanase | *Trichoderma viride* | Megazyme | 4.5 | 40 | 7 | 15 |
| 4-MUB-β-D-Xylopyranoside | β-xylosidase | *Bacillus subtilis* 168 | Prozomix | 7 | >35 | 5 | 10^-4^ |
| Starch Azure | α-amylase | *Aspergillus oryzae* | Sigma | 6.9 | 20 | 67 | 0.02 |
| 4-MUB-α-D-Glucopyranoside | α-glucosidase | *Saccharomyces cerevisiae* | Sigma | 6.8 | 37 | 0.3 | 10^-5^ |
| Chitin Azure | Chitinase | *Streptomyces griseus* | Sigma | 6 | 25 | 1000 | 0.5 |
| 4-MUB-N-β-D-Glucosaminidase | β-N-Acetylglucosaminidase | *Xanthomonas manihotis* | New England Biolabs | 6 | 37 | 5 | 10 |
| L-3,4-dihydroxyphenylalanine | Tyrosinase | Mushroom | Sigma | 6.5 | 25 | 50 | n.a. |

n.a. Not available

MUB Methylumbelliferyl

**Table S3.**  Fosmid clones sequenced in this study. Activities observed in each clone are listed by screening substrate in the substrates columns. Clones in which no GH genes corresponding to the detected activity could be identified were subjected to secondary screening against the respective substrates (+ or -). Clones in which genes were identified were not screened in the secondary screening (NA).

| **Clone**  **No.** | **GenBank Accession Number** | **Substrates** |  |  | **Secondary screening** |
| --- | --- | --- | --- | --- | --- |
| 1 | KF524439- KF524444 | 4-MUB- β-N-acetylglucosamine |  |  | NA |
| 2 |  | 4-MUB- β-D-Xyloside |  |  | - |
| 3 | KF524445 | 4-MUB- β-D-Xyloside |  |  | NA |
| 4 | KF524446 | Skim milk |  |  | NA |
| 5 | KF524447- KF524450 | 4-MUB- β-D-Cellobiose and PNP-β-D-Glucoside | |  | NA |
| 6 | KF524451- KF524453 | 4-MUB- β-D-Cellobiose, 4-MUB- β-D-Xyloside and PNP-β-D-Glucoside | | | NA |
| 7 |  | 4-MUB-α-D-Glucoside |  |  | - |
| 8 | KF524454- KF524457 | 4-MUB-α-D-Glucoside |  |  | + |
| 9 | KF524458- KF524460 | 4-MUB- β-D-Cellobiose |  |  | NA |
| 10 | KF524461- KF524465 | 5-Bromo-4-chloro-3-indolyl phosphate |  |  | NA |
| 11 | KF524466- KF524471 | 4-MUB- β-D-Cellobiose and PNP-β-D-Glucoside | |  | + |
| 12 | KF524472- KF524477 | 4-MUB- β-D-Cellobiose |  |  | NA |
| 13 | KF524478- KF524483 | 4-MUB- β-D-Cellobiose and PNP-β-D-Glucoside | |  | NA |
| 14 | KF524484- KF524488 | 4-MUB- β-D-Xyloside and PNP-β-D-Glucoside | |  | NA |
| 15 |  | 5-Bromo-4-chloro-3-indolyl phosphate |  |  | - |
| 16 | KF524489- KF524493 | 5-Bromo-4-chloro-3-indolyl phosphate |  |  | NA |
| 17 | KF524494- KF524496 | 4-MUB- β-D-Cellobiose |  |  | NA |
| 18 | KF524497- KF524501 | 5-Bromo-4-chloro-3-indolyl phosphate |  |  | NA |
| 19 | KF524502- KF524509 | 4-MUB-α-D-Glucoside |  |  | NA |
| 20 | KF524510- KF524512 | 4-MUB- β-D-Cellobiose |  |  | NA |
| 21 | KF524513- KF524515 | 4-MUB- β-N-acetylglucosamine |  |  | NA |
| 22 | KF524516- KF524517 | 4-MUB- β-D-Xyloside |  |  | NA |
| 23 | KF524518- KF524519 | Starch Azure |  |  | + |
| 24 | KF524520- KF524525 | AZCL Xylan and 4-MUB- β-D-Xyloside | |  | NA |
| 25 | KF524526 | 4-MUB- β-N-acetylglucosamine |  |  | NA |
| 26 | KF524527- KF524531 | 4-MUB- β-D-Cellobiose |  |  | NA |
| 27 |  | 4-MUB- β-N-acetylglucosamine |  |  | - |
| 28 | KF524532- KF524536 | 5-Bromo-4-chloro-3-indolyl phosphate |  |  | NA |
| 29 | KF524537- KF524542 | 4-MUB- β-D-Xyloside and PNP-β-D-Glucoside | |  | NA |
| 30 | KF524543- KF524551 | AZCL Xylan and 4-MUB- β-D-Cellobiose | |  | NA |
| 31 | KF524552- KF524555 | 4-MUB- β-D-Xyloside |  |  | NA |
| 32 | KF524556- KF524558 | AZCL Xylan |  |  | NA |
| **Clone** | **GenBank Accession Number** | **Substrates** |  |  | **Secondary screening** |
| 33 | KF524559 | 5-Bromo-4-chloro-3-indolyl phosphate |  |  | NA |
| 34 | **-** | 4-MUB- β-N-acetylglucosamine |  |  | - |
| 35 | KF524560- KF524565 | 4-MUB-α-D-Glucoside |  |  | + |
| 36 | KF524566- KF524568 | AZCL Xylan, 4-MUB- β-D-Cellobiose and 4-MUB- β-D-Xyloside | | | NA |
| 37 | KF524569- KF524574 | 4-MUB- β-D-Cellobiose, 4-MUB- β-D-Xyloside and PNP-β-D-Glucoside | | | + |
| 38 | KF524575- KF524580 | 4-MUB- β-D-Cellobiose and 4-MUB- β-D-Xyloside | |  | NA |
| 39 | KF524581 | 4-MUB- β-N-acetylglucosamine |  |  | NA |
| 40 | KF524582- KF524584 | Starch Azure |  |  | NA |
| 41 | KF524585- KF524592 | 4-MUB- β-D-Xyloside |  |  | NA |
| 42 | KF524593- KF524600 | 4-MUB-α-D-Glucoside |  |  | NA |
| 43 | KF524601- KF524607 | AZCL Xylan |  |  | + |
| 44 | KF524608- KF524610 | 4-MUB- β-D-Xyloside |  |  | NA |
| 45 | KF524611- KF524617 | Skim milk |  |  | NA |
| 46 | KF524618- KF524620 | 4-MUB- β-N-acetylglucosamine |  |  | NA |
| 47 | KF524621- KF524624 | 4-MUB-α-D-Glucoside |  |  | + |
| 48 | KF524625- KF524634 | AZCL Xylan and 4-MUB- β-D-Cellobiose | |  | NA |
| 49 | KF524635- KF524638 | 4-MUB- β-D-Xyloside |  |  | NA |
| 50 | KF524639- KF524643 | 4-MUB- β-D-Xyloside |  |  | NA |
| 51 | KF524644- KF524645 | AZCL Xylan |  |  | NA |
| 52 | KF524646- KF524647 | AZCL Xylan, 4-MUB- β-D-Cellobiose and 4-MUB- β-D-Xyloside | | | NA |
| 53 | KF524648- KF524652 | 4-MUB- β-D-Xyloside |  |  | NA |
| 54 | KF524654- KF524657 | 4-MUB- β-D-Xyloside |  |  | NA |
| 55 | KF524658 | 4-MUB- β-D-Xyloside |  |  | NA |
| 56 |  | 4-MUB-α-D-Glucoside |  |  | - |
| 57 |  | 4-MUB- β-N-acetylglucosamine |  |  | - |
| 58 |  | 4-MUB- β-N-acetylglucosamine |  |  | - |
| 59 | KF524659- KF524663 | 4-MUB- β-D-Cellobiose and 4-MUB- β-D-Xyloside | |  | NA |
| 60 | KF524664 | 4-MUB- β-N-acetylglucosamine |  |  | NA |
| 61 | KF524665-70 | 4-MUB- β-D-Cellobiose |  |  | NA |
| 62 |  | 4-MUB- β-D-Xyloside |  |  | - |
| 63 | KF524671- KF524673 | 4-MUB- β-D-Xyloside |  |  | NA |
| 64 | KF524674- KF524677 | 4-MUB- β-N-acetylglucosamine |  |  | NA |
| 65 | KF524678- KF524682 | AZCL Xylan and 4-MUB- β-D-Cellobiose | |  | NA |
| 66 | KF524683- KF524686 | AZCL HE-Cellulose |  |  | NA |
| 67 | KF524687- KF524693 | AZCL HE-Cellulose |  |  | NA |
| 68 | KF524694- KF524699 | AZCL Xylan and 4-MUB- β-D-Cellobiose | |  | NA |
| 69 | KF524700- KF524707 | AZCL HE-Cellulose and 4-MUB- β-D-Cellobiose | |  | NA |
| 70 | KF524708- KF524710 | AZCL Xylan and 4-MUB- β-D-Cellobiose | |  | NA |
| 71 | KF524711- KF524719 | 5-Bromo-4-chloro-3-indolyl phosphate |  |  | NA |
| 72 | KF524720- KF524722 | 4-MUB- β-D-Xyloside |  |  | + |
| 73 | KF524723- KF524728 | AZCL Xylan |  |  | NA |
| **Clone** | **GenBank Accession Number** | **Substrates** |  |  | **Secondary screening** |
| 74 | KF524729- KF524735 | AZCL Xylan and 4-MUB- β-D-Cellobiose | |  | NA |
| 75 | KF524736- KF524737 | 4-MUB- β-D-Cellobiose |  |  | NA |
| 76 | KF524738- KF524743 | Skim milk |  |  | NA |
| 77 | KF524744- KF524748 | AZCL Xylan |  |  | NA |
| 78 | KF524749- KF524752 | AZCL Xylan, 4-MUB- β-D-Cellobiose and 4-MUB- β-D-Xyloside | | | NA |
| 79 | KF524753- KF524756 | 5-Bromo-4-chloro-3-indolyl phosphate |  |  | NA |
| 80 | KF524757- KF524762 | 4-MUB- β-D-Xyloside |  |  | NA |
| 81 | KF524763- KF524769 | 4-MUB- β-D-Xyloside and PNP-β-D-Glucoside | |  | NA |
| 82 | KF524770- KF524773 | PNP-β-D-Glucoside |  |  | NA |
| 83 | KF524774- KF524775 | 5-Bromo-4-chloro-3-indolyl phosphate |  |  | NA |
| 84 | KF524776- KF524780 | 4-MUB- β-D-Cellobiose, 4-MUB- β-D-Xyloside and PNP-β-D-Glucoside | | | NA |
| 85 | KF524781- KF524787 | Starch Azure |  |  | NA |
| 86 | KF524788- KF524793 | AZCL Xylan |  |  | NA |
| 87 | KF524794- KF524801 | AZCL Xylan and 4-MUB- β-D-Cellobiose | |  | NA |
| 88 | KF524802- KF524808 | 5-Bromo-4-chloro-3-indolyl phosphate |  |  | NA |
| 89 | KF524809- KF524815 | 4-MUB- β-N-acetylglucosamine |  |  | NA |
| 90 |  | Starch Azure |  |  | - |
| 91 | KF524816- KF524822 | AZCL Xylan, 4-MUB- β-D-Cellobiose and 4-MUB- β-D-Xyloside | | | NA |
| 92 | KF524823- KF524827 | 4-MUB- β-N-acetylglucosamine |  |  | NA |
| 93 | KF524828- KF524833 | 4-MUB- β-D-Cellobiose |  |  | NA |
| 94 | KF524834 | 4-MUB- β-N-acetylglucosamine |  |  | + |
| 95 | KF524835- KF524837 | Starch Azure |  |  | NA |

**Supplementary figures**


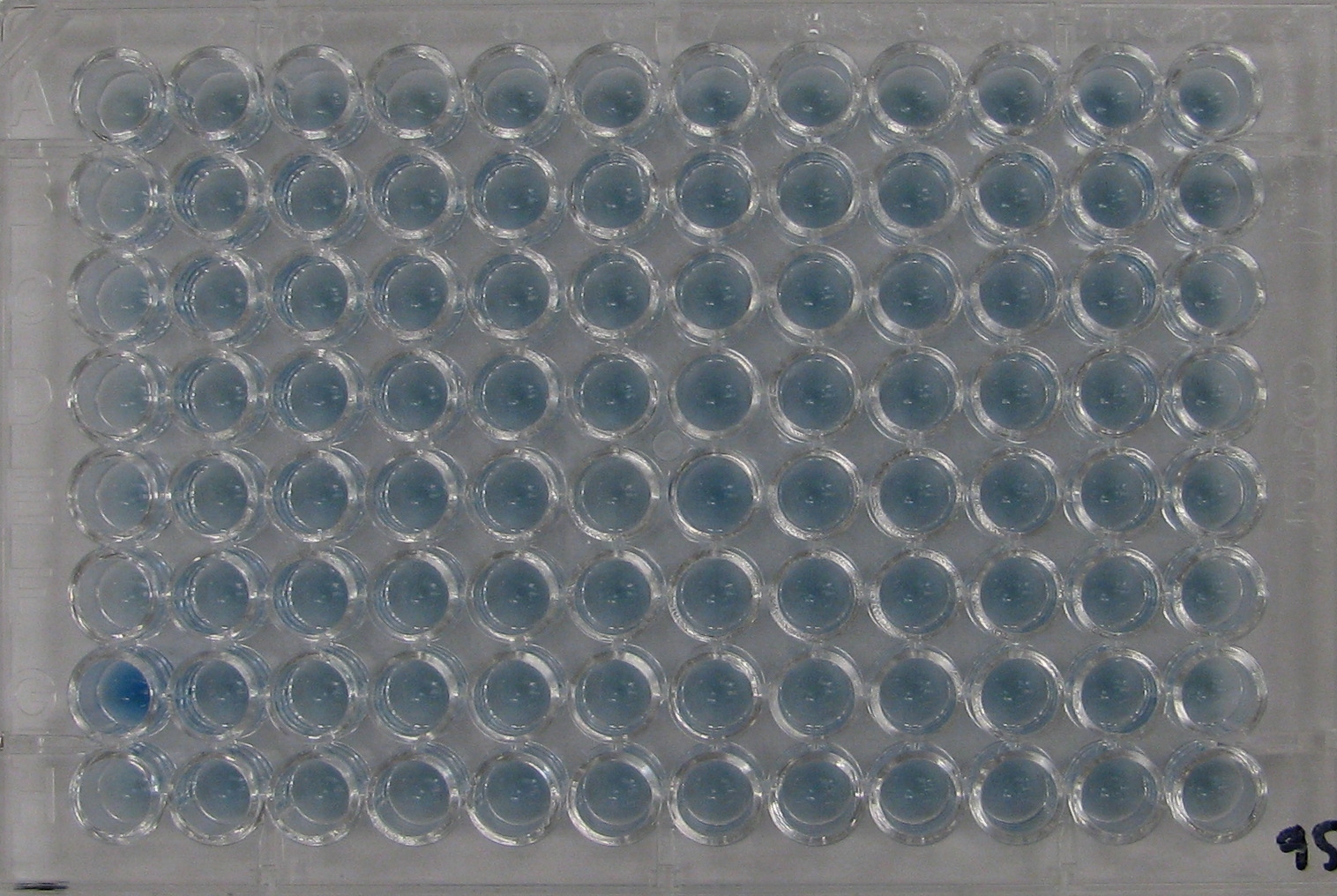

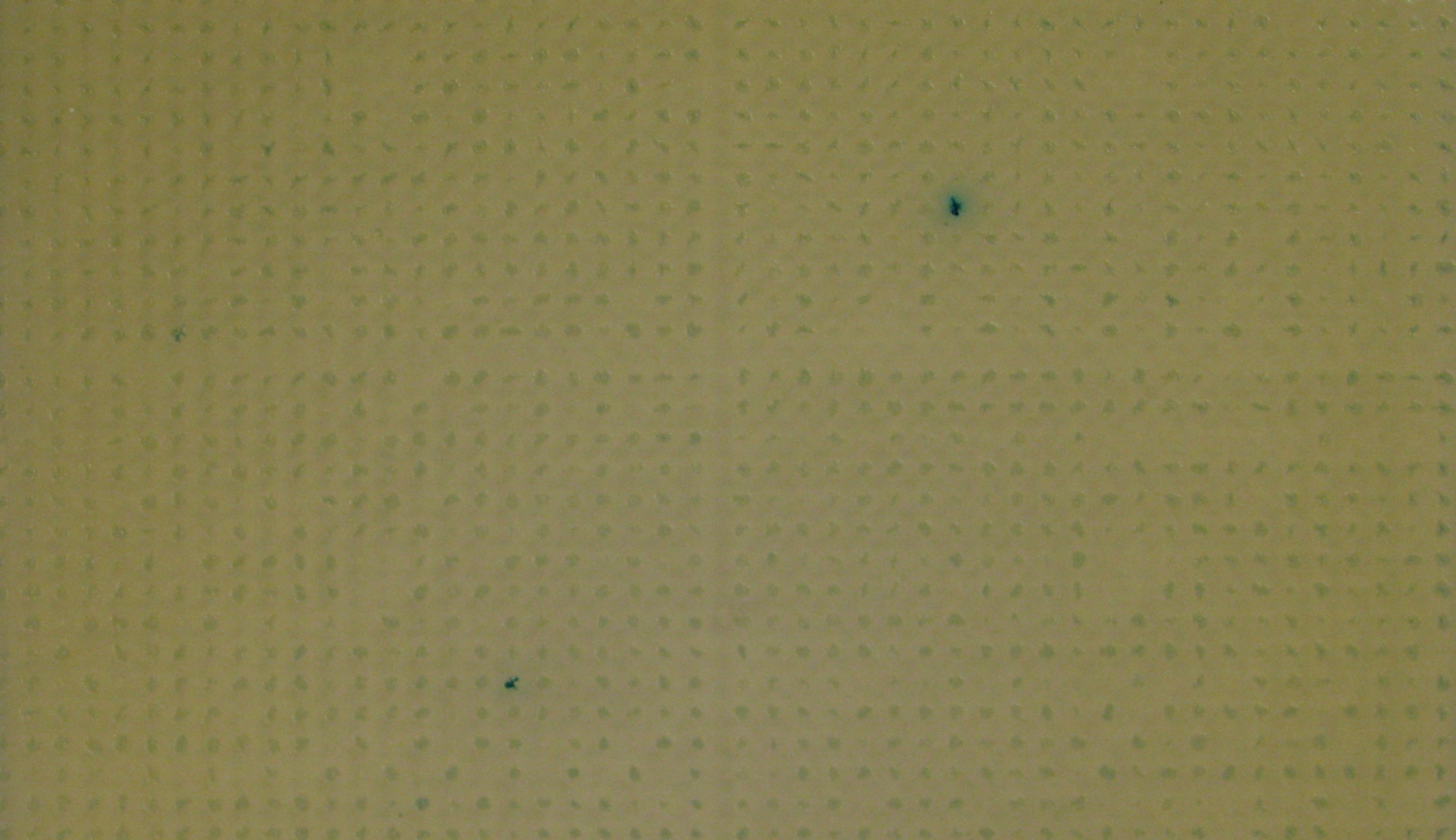


c)

b)

a)

**Figure S1.** Starch degradation (a) and phosphatase assays (b) using the *Escherichia coli* EPI300-T1R screening host modified with gene-specific knockouts to reduce wild-type α-amylase, α-glucosidase and phosphatase activities.


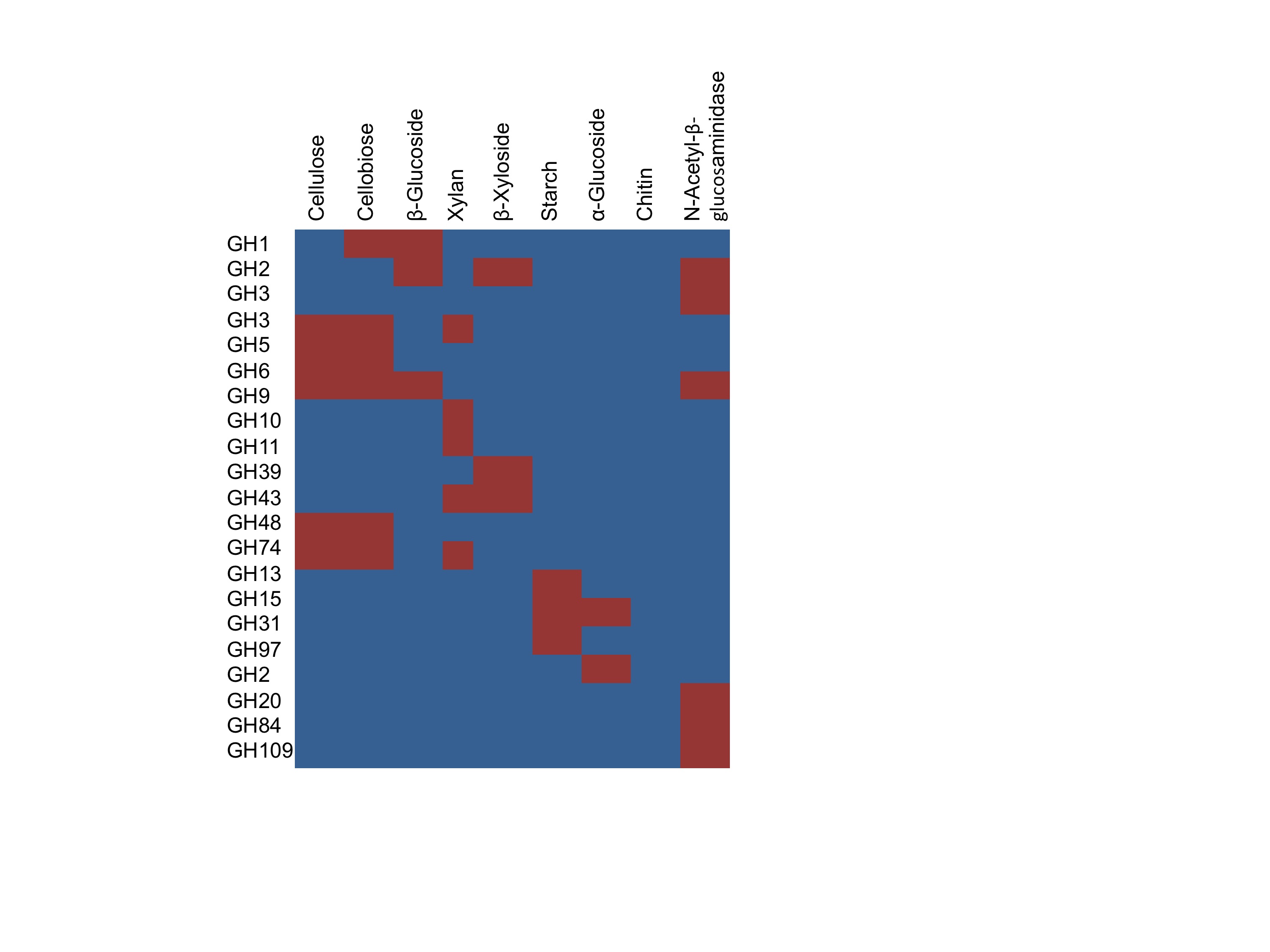


**Figure S2.** *In silico* functional annotation of glycoside hydrolase genes identified from fosmid clones. The matrix is based on known activities (red bars) reported for the glycoside hydrolase families in the Carbohydrate-active enzymes database.

a)

b)

c)

d)

e)

f)

g)

**Figure S3.** Taxonomic assignment of tetranucleotide frequency bins using conserved phylogenetic marker genes identified from the bins. a) DNA gyrase B subunit, b) Translation elongation factor Tu, c) Translation elongation factor P, d) Recombinase A, e) 50S ribosomal protein L31, f) 30S ribosomal protein S2, g) 30S ribosomal protein S21. Phylogenetic marker genes were not identified from all bins.
